# Enantiomeric Separation of New Chiral Azole Compounds

**DOI:** 10.3390/molecules26010213

**Published:** 2021-01-04

**Authors:** Marziyeh E. Kenari, Joshua I. Putman, Ravi P. Singh, Brandon B. Fulton, Huy Phan, Reem K. Haimour, Key Tse, Alain Berthod, Carl J. Lovely, Daniel W. Armstrong

**Affiliations:** 1 Department of Chemistry and Biochemistry, University of Texas at Arlington, Arlington, TX 76016, USA; marziyeh.eshaghikenari@mavs.uta.edu (M.E.K.); joshua.putman@mavs.uta.edu (J.I.P.); ravi.singh@mavs.uta.edu (R.P.S.); brandon.fulton@mavs.uta.edu (B.B.F.); huy.phan2@mavs.uta.edu (H.P.); reem.haimour@mavs.uta.edu (R.K.H.); key.tse@mavs.uta.edu (K.T.); lovely@uta.edu (C.J.L.); 2 Institut des Sciences Analytiques, CNRS, Université of Lyon, 5 rue de la Doua, 69100 Villeurbanne, France; berthod@univ-lyon1.fr

**Keywords:** oxazole, thiazole, pyridine, enantiomer separation, mobile phase modes, macrocyclic glycopeptides, core-shell supports

## Abstract

Twelve new azole compounds were synthesized through an ene reaction involving methylidene heterocycles and phenylmaleimide, producing four oxazoles, five thiazoles, and one pyridine derivative, and ethyl glyoxylate for an oxazole and a thiazole compound. The twelve azoles have a stereogenic center in their structure. Hence, a method to separate the enantiomeric pairs, must be provided if any further study of chemical and pharmacological importance of these compounds is to be accomplished. Six chiral stationary phases were assayed: four were based on macrocyclic glycopeptide selectors and two on linear carbohydrates, i.e., derivatized maltodextrin and amylose. The enantiomers of the entire set of new chiral azole compounds were separated using three different mobile phase elution modes: normal phase, polar organic, and reversed phase. The most effective chiral stationary phase was the MaltoShell column, which was able to separate ten of the twelve compounds in one elution mode or another. Structural similarities in the newly synthesized oxazoles provided some insights into possible chiral recognition mechanisms.

## 1. Introduction

Heterocyclic fragments or moieties are present in the majority of marketed active pharmaceutical ingredients (APIs) [1]. The reason for the prevalence of such heteroatom- and especially nitrogen-containing rings in drugs is their similarity to natural biologically active compounds. Heterocyclic moieties present in many APIs provide strong and specific points for a variety of intermolecular interactions. In addition, they afford a degree of rigidity that can enhance chiral recognition in stereoselective processes. Another aspect of the heterocyclic moiety is to influence API polarity and hydrophobicity, which can affect its water solubility [1]. It is well established that nitrogen-containing heterocyclic compounds are key players in many biochemical reactions and are present in API structures [2].

Pyridines, oxazoles, and thiazoles are aromatic *N*-containing heterocycles with a six-membered ring with a single N atom, five-membered rings with both O and N heteroatoms, and S and N heteroatoms, respectively [3,4]. The copper-catalyzed hydroamination of propargyl imidates was recently proposed as an efficient way to obtain alkyl-or aryl-substituted dihydrooxazoles providing interesting intermediates for further elaboration [5]. Recent investigations from our laboratory have focused on the synthesis and reactivity of “pre-aromatic” heterocycles derived from propargylic systems [6,7]. A subsequent reaction with these methylidene compounds can take advantage of aromatization as a driving force. In this context, an “ene” reaction [8] has been used to forge a new C-C bond, which, when coupled with a prochiral enophile, results in the formation of a new chiral center [9]. Following this procedure, we prepared twelve new *N*-containing heterocyclic compounds reacting *N*-phenylmaleimide or ethyl-glyoxylate with methylidene oxazolines or thiazolines. Upon the addition reaction, carbon 3 of the substituted N-phenylsuccinimide, or the secondary alcohol obtained with ethyl-glyoxylate, became stereogenic centers. In the present iteration, the reaction produced racemic mixtures of the N-containing heterocycles. The long-term goal of this investigation is to develop asymmetric variants.

Today, it is difficult to submit a new racemic API to regulatory agencies due to possible differences in the biological effects of the two enantiomers [10]. In drug discovery, the two enantiomers are needed for testing since it is not immediately known which one or both will become API(s). Hence, it is of primary importance to be able to separate the enantiomers of the newly prepared heterocyclic compounds. Since all aromatic heterocyclic compounds are strong UV-absorbing molecules, they can be detected easily with basic HPLC. In enantioselective HPLC, the chiral selector is most often attached to the stationary phase support. Enantiomeric separations will only be possible if the appropriate chiral stationary phase is used [11,12,13,14,15,16,17,18,19,20,21,22,23]. It is the aim of this work to present the chromatographic separation of the enantiomers of twelve newly synthesized N-containing heterocyclic compounds.

## 2. Materials and Methods

### 2.1. Chemicals

Heptane, hexane, methanol, ethanol, and acetonitrile were the HPLC grade solvents obtained from Fisher Scientific (Fair Lawn, NJ, USA) and used as received. Acetic acid, triethylamine, and ammonium formate were purchased from Sigma-Aldrich (Millipore-Sigma, Burlington, MA, USA).

**Scheme 1:**
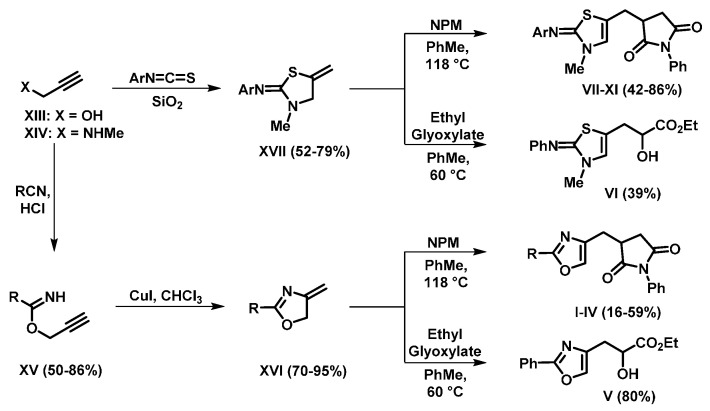
Synthetic routes for the new chiral azoles studied [23].

As outlined in Scheme 1, racemic analytes were prepared via short two and three-step sequences beginning with readily available propargylic derivatives XIII and XIV. In the case of the oxazole derivatives I-V, ene substrates were prepared through reaction of propargyl alcohol (XIII) with the appropriate nitrile derivative in the presence of HCl, affording the corresponding imidate XV in good-to-excellent yield [5]. Treatment of the imidates with CuI resulted in hydroamination of the alkyne and formation of the methylidene oxazoles XVI [5]. Exposure of XVI to either *N*-phenylmaleimide or ethyl glyoxylate delivered the corresponding ene adducts I-V in modest to good yields [24]. The thiazoline derivatives XVII were prepared in analogous fashion through a one-step thio acylation/hydroamination sequence [6,7] and then subjected to ene reactions with *N*-phenylmaleimide and ethyl glyoxylate, which afforded the corresponding adducts in generally good yields.

### 2.2. Chromatography

The liquid chromatography system used was the 1220 Infinity II set from Agilent (Santa Clara. CA. USA) including a quaternary pump, mobile phase degasser, 96 vial sample injector, column thermostat, and diode array UV detector. A personal computer drove the chromatographic system and handled data with the OpenLab CDS ChemStation software (Agilent). Acetonitrile solutions of all racemic samples were made at a concentration of 2 mg/mL. One microliter of each individual solution was injected for each analysis.

Table 1 lists the characteristics of the chiral columns used [11,12,13,14,15,16,17,18,19,20,21,22,23]. Except for the NicoShell column (3 mm i.d.), the AZYP columns were all 4.6 mm internal diameter columns packed with superficially porous (SPP) 2.7 µm particles provided by AZYP, LLC (Arlington, TX, USA). The Chiralpack IA -3 column (also 4.6 mm i.d.) was packed with 3 µm fully porous particles and provided by Daicel (Chiral Technologies, West Chester, PA, USA).

## 3. Results and Discussion

### 3.1. Novel Azole Compounds

**Figure 1:**
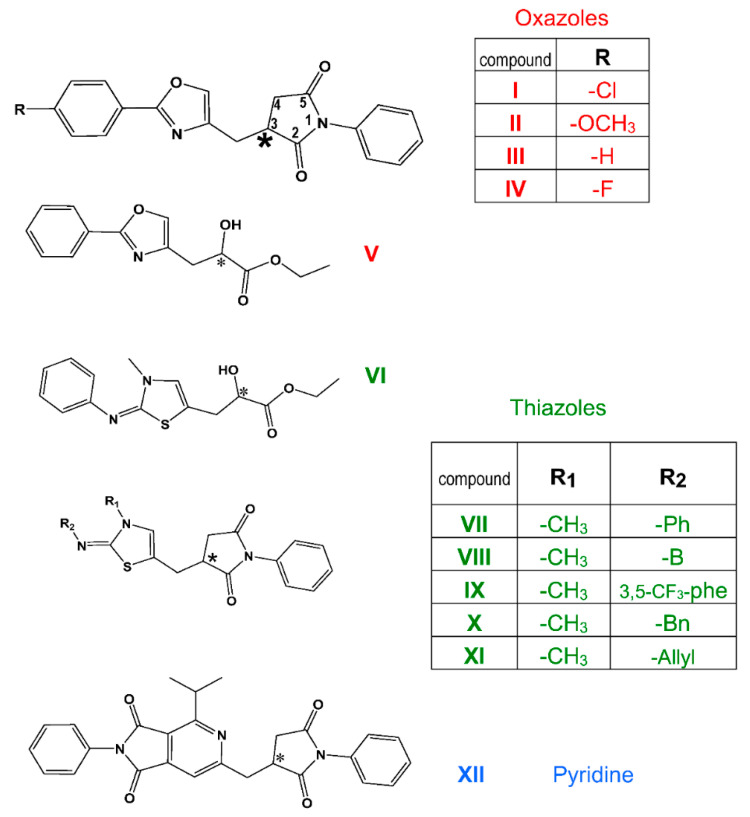
Newly synthesized azole compounds. I-IV and VII-XII are *N*-phenylmaleimide adducts; V and VI are ethylglyoxylate adducts. Oxazoles I-V in red; thiazoles VI-XI in green. Code: * stereogenic center; -Ph: phenyl group; -Bn: -CH_2_-Ph or benzyl group; -allyl: -CH_2_-CH=CH_2_ group.0.

A degassed solution of toluene containing *N*-phenylmaleimide or ethyl glyoxylate reacted with the corresponding oxazoline [5] and thiazoline [6] methylidenes was heated at reflux overnight and resulted in an ene reaction providing twelve new chiral azole compounds. The detailed processes and reactions are presented elsewhere [24]. Ten compounds, coded I-IV and VII-XII, were N phenylmaleimide adducts having a stereogenic center on carbon 3 of the succinimide ring (Figure 1). Only two compounds, V and VI, were ethyl glyoxalate adducts with a secondary alcohol stereogenic center (starred in Figure 1).

### 3.2. Enantiomer Separation of Chiral Azoles

Separations of the azole enantiomers were investigated with six different chiral stationary phases (CSPs) as listed in Table 1, using three different mobile phase modes: (i) the reversed phase (RP) mode where the polar mobile phases are mixtures of an aqueous buffer and a miscible polar organic solvent (methanol or acetonitrile); (ii) the polar organic (PO) mode where the mobiles phases are waterless mixtures of polar organic solvents possibly with small amounts of triethylamine and acetic acid; (iii) the normal phase (NP) mode that uses a nonpolar solvent, mainly heptane, containing a small amount of an alcohol, either ethanol or isopropanol to adjust polarity and retention.

The chromatographic parameters considered were the retention times of each enantiomer, allowing one to calculate the corresponding retention factors, *k*_1_ and *k*_2_, using the column dead time, *t_o_,* determined as the first UV detector variation after injection:

*k* = (*t_r_* − *t_o_*)/*t_o_* (1)

The enantioselectivity factor, α, is the ratio of the two enantiomer retention factors, *k*_2_/*k*_1_, and the resolution factor, *Rs*, is defined as

(2) $$Rs=\frac{2(t_{2}-t_{1})}{W_{1}+W_{2}}$$

where *W_i_* is the peak width of peaks 1 and 2 at peak base, respectively. A resolution factor of 1.5 corresponds to the signal returning to baseline between separated peaks. A *Rs* higher than 1.5 corresponds to fully separated peaks with space between, and a *Rs* smaller than 1.5 corresponds to partially merged peaks. If *Rs* is equal or smaller than 0.4, the two peaks are not distinguishable and only a broadened single peak is observed.

**Figure 2:**
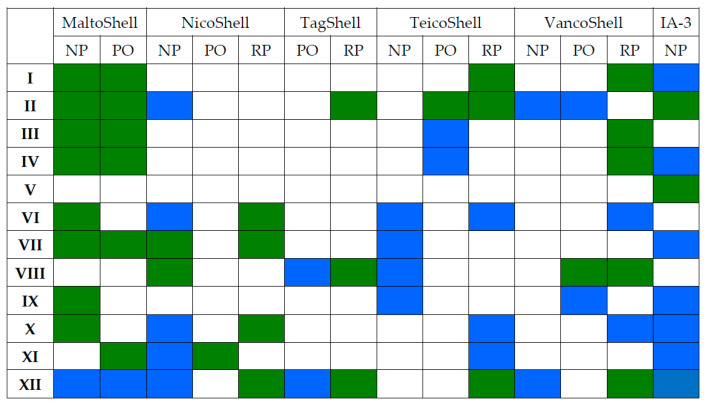
Heat map or score card of the separation of the enantiomers of the 12 azole compounds on the Table 1 columns with the different mobile phase modes. NP: normal mobile phase; RP: reversed polar aqueous mobile phase; PO: polar organic waterless mobile phase. Green color: *Rs* > 1.5; blue color: 0.4 < *Rs* < 1.5; no color: *Rs* < 0.4.

Figure 2 presents the results for the set of 12 azole compounds in the form of a heat map. For each column and each mobile phase mode, the color indicates the level of separation: green corresponds to a full enantiomeric separation (*Rs* > 1.5), blue indicates a partial separation (0.4 < *Rs* < 1.5), and no color is for no visible separation (*Rs* < 0.4). The heat map or score card shows that the MaltoShell chiral stationary phase provides a baseline separation for 10 of the 12 azole enantiomers followed by the NicoShell, with 6 separated out of 12, and the VancoShell (with 5 of 12). In the normal phase mode alone, the MaltoShell column separated 9 azoles or 75% of the set of 12. It could separate seven pairs in the polar organic mode. The IA-3 column also could separate nine enantiomeric pairs but with a lower resolution, although the PO and RP mode were not assayed with this column.

**Figure 3:**
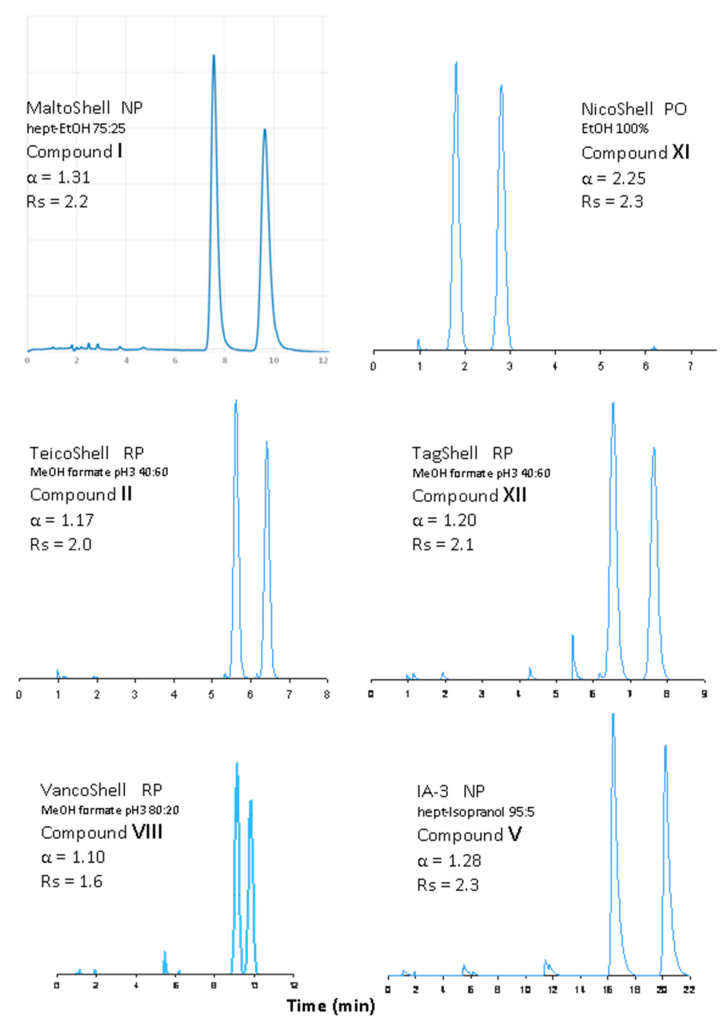
Examples of separation of the enantiomers of the Figure 1 chiral azoles on each of the Table 1 chiral columns. Mobile phase flow rate: 1 mL/min, 30 °C, detection UV 254 nm.

The TeicoShell column could separate in one mobile phase mode or another, at least partially, 11 of the 12 compounds tested. Similarly, the VancoShell and the NicoShell columns could separate, respectively, 9 and 7 compounds in the different mobile phase modes (Figure 2). The TagShell column was the least effective for this particular set of chiral compounds. Table 2 lists the optimal chromatographic conditions and chiral stationary phases used to obtain the best separations for each of this set of chiral azole compounds (Figure 1). Figure 3 presents a selection of chromatograms obtained on each of the Table 1 columns using different mobile phase modes.

### 3.3. Chiral Mechanism Considerations

Chirality in these azole compounds is the result of four different substituents attached to the same sp^3^ hybridized carbon atom, which is referred to as the stereogenic center (stared in Figure 1). Compounds II and XII were the only ones that were separated by all chiral stationary phases used (Figure 2). Compound II has its stereogenic center as part of the succinimide ring between two rigid carbonyl substituents, the third substituent is a small hydrogen atom, and the last substituent is a large phenyl substituted oxazole with a methoxy group in the para position (Figure 1). Compound XII is the only pyridine-containing azole of the set. The arrangement of its stereogenic center is the same as that of Compound II for the three first substituents, with the fourth substituent being the large pyridine ring rigidly fused with the *N*-phenyl maleimide moiety. Rigid structures are generally easier to differentiate by chiral selectors [25].

Conversely, compound V was not separated by any of the macrocyclic SPP CSPs (Table 2 and Figure 2). Only the Chiralpack IA-3 column could separate these enantiomers. Compound V is the only example of an oxazole obtained by reacting with ethyl glyoxylate. The stereogenic center obtained is an exocyclic secondary alcohol. It has two small substituents: a hydrogen atom and a hydroxyl group, and two larger substituents: a 2-phenyloxazole group and an ethyl carboxylate group. All four groups attached to the stereogenic center are free to rotate and/or are quite flexible, which results in a much less rigid structure more difficult to differentiate. However, thiazole compound VI, which is derived from ethyl glyoxylate as well, also has an exocyclic alcohol substituent; thus, three of the four substituents on the stereogenic center are identical to compound V. The fourth substituent is a more rigid, angular, and slightly larger substituted thiazole. It also has an additional tertiary amine group (Figure 1). This single substituent renders compound VI enantiomers separable by all of the macrocyclic SPP chiral stationary phases, but oddly enough, not by the IA-3 stationary phase, in the same conditions that separated Compound V.

**Figure 4:**
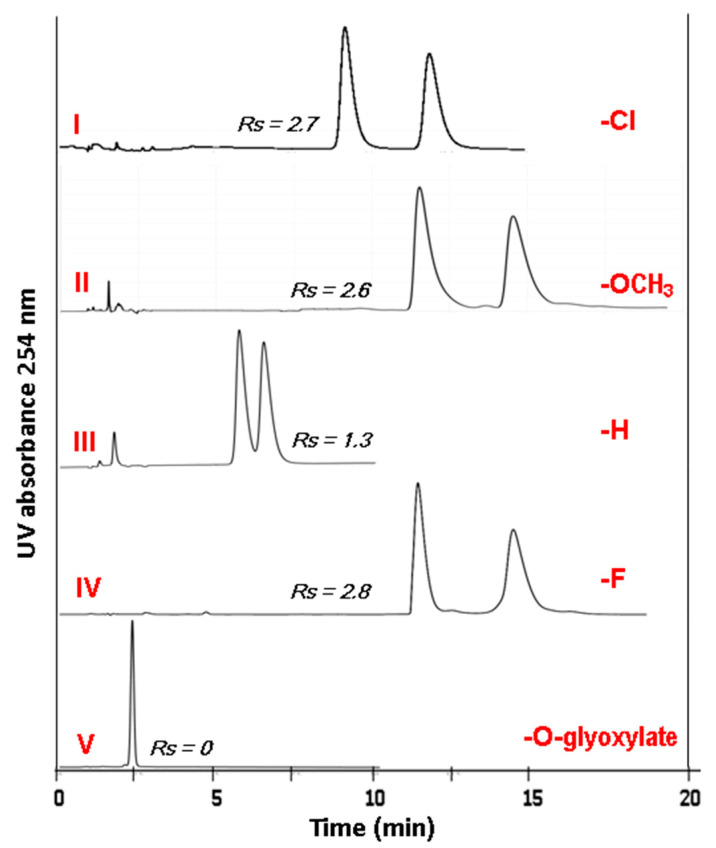
Chromatograms of the five oxazole compounds obtained on the MaltoShell chiral column, all eluted with the same heptane-ethanol 80:20 % *v*/*v* normal mobile phase; 30 °C, 1 mL/min, UV 254 nm. See Table 1 for compound codes; the structural difference is pointed out on the right.

Figure 4 combines the chromatograms of all the azole compounds obtained using the MaltoShell chiral stationary phase in the normal phase mode with an identical heptane-ethanol, 80:20 % *v*/*v* mobile phase. It should be noted that this is not the optimal mobile phase for all these compounds, but it does allow comparison under identical experimental conditions. The bold red color text on the right is used to denote the chemical groups differentiating these oxazoles. Oxazoles I to IV have a similar structure; they only differ in the substituent at the para position of the oxazole aryl group (Figure 1). Using identical chromatographic conditions, compounds I, II, and IV chromatograms are similar, but that of compound III, which lacks a para substituent, is different, eluting faster with a significantly lower enantioresolution factor (Figure 4). This means that electron-withdrawing substituents, either halogens (I and IV) or a methoxy group (II), increase the retention and improve chiral recognition of these oxazoles by the maltodextrin chiral selector.

Thiazoles VII to XI also have similar structures, but very different results were obtained under similar or even identical chromatographic conditions. No definitive conclusions on chiral recognition of thiazoles can be obtained with these results, but all six chiral thiazoles could have their enantiomers fully separated (*Rs* > 1.5) by one column or another (Figure 2 and Table 2).

## 4. Conclusions

Twelve newly synthesized racemic azole compounds have not been resolved previously by any means, until now. Additionally, such compounds are known to be pharmacologically active. Such enantiomeric separations are most expeditiously accomplished by direct chiral separation techniques. Using six chiral stationary phases with different selectors, it was possible to fully separate the enantiomers of the entire set of azole compounds. Some insights into the azole chiral recognition mechanism could be obtained by comparing the separation results obtained under identical chromatographic conditions with compounds having similar structures. The oxazole recognition ability by the MaltoShell chiral selector was very sensitive to the presence of an electron-withdrawing group on its phenyl substituent, even though this group was far from the stereogenic center. Indeed, both retention and resolution factors of the enantiomeric pairs of the rigid chiral oxazoles were affected. Such an observation was not possible in the limited case of the chiral thiazoles examined.

## Figures and Tables

**Table 1:** Characteristics of the 4.6 mm i.d. chiral columns used in this work and the 3.0 mm i.d. NicoShell column. ^a^

Brand Name	Length (cm)	Selector	Screening Mode	Supplier	Ref.
MaltoShell	10	Derivatized maltodextrin	NP, PO	AZYP LLC	[11]
NicoShell	15	Modified glycopeptide	NP, PO, RP	AZYP LLC	[19]
TagShell	10	Teicoplanin aglycone	PO, RP	AZYP LLC	[20]
TeicoShell	10	Teicoplanin glycopeptide	NP, PO, RP	AZYP LLC	[20]
VancoShell	15	Vancomycin glycopeptide	NP, PO, RP	AZYP LLC	[20]
ChiralPack IA-3	15	Amylose-3,5-dimethylphenyl carbamate	NP	Daicel	[22]

^a^ NP: normal phase mode with alkane/alcohol mobile phases; PO: waterless polar organic mode with alcohol or acetonitrile-buffer mobile phases; RP: reversed-phase mode with alcohol/aqueous buffer mobile phases. All AZYP columns were packed with 2.7 µm core shell superficially porous particles. The Chiralpack column was packed with 3 µm fully porous particle.

**Table 2:** Selected enantiomer separations of azole compounds whose structures are shown in Figure 1. ^a^

Comp	Column	Mobile Phase % *v*/*v*	Flow mL/min	Temp. °C	t_r1_ min	t_r2_ min	α	*Rs*
**I**	MaltoShell	NP—Hep-EtOH 80:20	1.0	40	9.1	11.9	1.34	2.7
	TeicoShell	RP—MeOH-formate pH 3, 50:50	0.8	40	10.0	11.3	1.14	2.2
	VancoShell	RP—AcN-formate pH 6 20:80	0.6	30	33.5	35.9	1.08	1.6
**II**	MaltoShell	NP—Hep-EtOH 80:20	1.0	30	11.5	14.5	1.28	2.9
	TagShell	RP—MeOH-formate pH 3, 40:60	0.6	40	6.6	7.2	1.11	2.6
	TeicoShell	RP—MetOH-formate pH 3, 40:60	0.6	40	5.6	6.4	1.17	2.0
**III**	MaltoShell	PO—AcN-MeOH-aa-tea 60:40:0.3:0.2	1.0	40	4.3	5.3	1.30	2.3
	VancoShell	RP—AcN-formate pH 6, 20:80	0.6	30	20.0	21.9	1.10	1.9
**IV**	MaltoShell	NP—Hep-EtOH 95:5	1.0	30	11.4	14.4	1.28	2.8
		PO—MeOH-EtOH 50:50	0.6	30	3.0	3.5	1.25	2.2
	VancoShell	RP—AcN-formate pH 6, 20:80	0.6	30	10.6	11.4	1.08	1.4
**V**	IA-3	NP—Hept-IPA 95:5	0.4	40	16.3	20.1	1.28	2.3
**VI**	MaltoShell	NP—Hept-EtOH 95:5	1.0	40	10.6	12.9	1.24	2.1
	NicoShell	RP—MeOH-formate pH 4, 30:70	1.0	50	8.2	8.8	1.08	1.4
**VII**	MaltoShell	RP—AcN-formate pH 3, 60:40	1.0	40	3.4	4.2	1.33	2.5
		NP—Hep-EtOH 80:20	1.0	40	22.1	30.7	1.38	2.5
**VIII**	NicoShell	NP—Hep-EtOH 90:10	1.0	30	30.4	35.2	1.16	2.2
	TagShell	RP—MeOH-formate pH 3, 40:60	0.6	50	5.5	6.4	1.20	2.4
	VancoShell	PO—MeOH-EtOH 25:75	0.6	30	34.8	37.8	1.08	1.6
		RP—AcN-formate pH 3, 20:80	1.0	30	9.1	9.8	1.10	1.6
**IX**	MaltoShell	NP—Hep-EtOH 80:20	1.0	30	4.8	5.6	1.21	1.4
		RP—MeOH-formate pH 6, 50:50	0.6	30	2.3	2.8	1.35	2.2
**X**	MaltoShell	NP—Hep-EtOH 80:20	1.0	30	4.7	5.4	1.19	1.4
	NicoShell	RP—AcN-formate pH 6, 85-15	1.0	30	17.6	20.9	1.20	4.5
**XI**	MaltoShell	NP—Hep-EtOH 80:20	1.0	30	6.2	12.5	2.21	3.9
	NicoShell	PO—EtOH 100	1.0	30	1.8	2.8	2.25	2.3
**XII**	MaltoShell	NP—Hep-EtOH 75:25	0.6	30	9.2	10.4	1.15	1.5
	TagShell	RP—MeOH-formate pH 3, 40:60	0.6	50	6.5	7.6	1.20	2.1
	TeicoShell	RP—MeOH-formate pH 3, 40:60	0.6	40	5.6	6.3	1.15	2.4
	VancoShell NicoShell	RP—MeOH-formate pH 4, 50:50 RP-MeOH-formate pH 4, 30:70	1.0 0.25	30 25	5.0 8.1	5.5 8.8	1.12 1.14	1.6 2.1

^a^ AcN: acetonitrile; EtOH: ethanol; formate: NH_4_HCOO ammonium formate buffer 20 mM in aqueous solution adjusted at indicated pH by formic acid; Hep: heptane; IPA: isopropanol; MeOH: methanol; aa: acetic acid; tea: triethylamine; t_r1_: retention time of the first eluting enantiomers; α: enantioselectivity factor; *Rs*: enantioresolution factor.

## Data Availability

More information on compound syntheses can be found in Ref. [23].

## References

[1] Gomtsyan A. (2012). Heterocycles in drugs and drug discovery. Chem. Heterocycl. Compd..

[2] Walsh C.T. (2015). Nature loves nitrogen heterocycles. Tetrahedron Lett..

[3] Hartner F.W., Katritzky A.R., Rees W.R., Scriven E.F.V. (1996). Oxazoles. Chemistry of Heterocyclic Compounds: A Series of Monographs.

[4] Dondoni A., Mermo P., Katritzky A.R., Rees W.R., Scriven E.F.V. (1996). Thiazoles. Chemistry of Heterocyclic Compounds: A Series of Monographs.

[5] Fricke P.J., Stasko J.L., Robbins D.T., Gardner A.C., Stash J., Ferraro M.J., Fennie M. (2017). Copper-catalyzed hydroamination of propargyl imidates. Tetrahedron Lett..

[6] Singh R.P., Gout D., Lovely C.J. (2019). Tandem Thioacylation-Intramolecular Hydrosulfenylation of Propargyl Amines - Rapid Access to 2-Aminothiazolidines. Eur. J. Org. Chem..

[7] Singh R.P., Aziz M.N., Gout D., Fayad W., El-Manawaty M.A., Lovely C.J. (2019). Novel thiazolidines: Synthesis, antiproliferative properties and 2D-QSAR studies. Bioorg. Med. Chem..

[8] Niu D., Hoye T.R. (2013). The aromatic ene reaction. Nat. Chem..

[9] Nalivela K.S., Rudolph M., Baeissa E.S., Alhogbi B.G., Mkhalid I.A.I., Hashmi A.S.K. (2018). Sequential Au/Cu Catalysis: A Two Catalyst One-Pot Protocol for the Enantioselective Synthesis of Oxazole α-Hydroxy Esters via Intramolecular Cyclization/Intermolecular Alder-Ene Reaction. Adv. Synth. Catal..

[10] Guida W.C., Daniel K.G. (2011). The Significance of Chirality in Drug Design and Development. Curr. Top. Med. Chem..

[11] Thakur N., Patel R.A., Talebi M., Readel E.R., Armstrong D.W. (2019). Enantiomeric impurities in chiral catalysts, auxiliaries and sythons used in enantioselective synthesis. Part 5. Chirality.

[12] Zhang X., Bao Y., Huang K., Barnett-Rundlett K.L., Armstrong D.W. (2009). Evaluation of dalbavancin as chiral selector for HPLC and comparison with teicoplanin-based chiral stationary phases. Chirality.

[13] Stalcup A., Chang S.-C., Armstrong D.W. (1991). Effect of the configuration of the substituents of derivatized β-cyclodextrin bonded phases on enantioselectivity in normal-phase liquid chromatography. J. Chromatogr. A.

[14] Hilton M., Armstrong D.W. (1991). Evaluation of a chiral crown ether LC column for the separation of racemic amines. J. Liq. Chromatogr..

[15] Ekborg-Ott K.H., Kullman J.P., Wang X., Gahm K., He L., Armstrong D.W. (1998). Evaluation of the macrocyclic antibiotic avoparcin as a new chiral selector for HPLC. Chirality.

[16] Péter A., Vékes E., Armstrong D.W. (2002). Effects of temperature on retention of chiral compounds on a ristocetin A chiral stationary phase. J. Chromatogr. A.

[17] Sun P., Armstrong D.W. (2010). Effective enantiomeric separations of racemic primary amines by the isopropyl carbamate-cyclofructan6 chiral stationary phase. J. Chromatogr. A.

[18] Patel D.C., Breitbach Z.S., Wahab M.F., Barhate C.L., Armstrong D.W. (2015). Gone in seconds: praxis, performance, and peculiarities of ultrafast chiral liquid chromatography with superficially porous particles. Anal. Chem..

[19] Patel D.C., Wahab M.F., Armstrong D.W., Breitbach Z.S. (2016). Advances in high-throughput and high-efficiency chiral liquid chromatographic separations. J. Chromatogr. A.

[20] Barhate C.L., Lopez D.A., Makarov A.A., Bu X., Morris W.J., Lekhal A., Hartman R., Armstrong D.W., Regalado E.L. (2018). Macrocyclic glycopeptide chiral selectors bonded to core-shell particles enables enantiopurity analysis of the entire verubecestat synthetic route. J. Chromatogr. A.

[21] Hellinghausen G., Lee J.T., Weatherly C.A., Lopez D., Armstrong D.W. (2017). Evaluation of nicotine in tobacco-free-nicotine commercial products. Drug Test. Anal..

[22] Barhate C.L., Wahab M.F., Breitbach Z.S., Bell D.S., Armstrong D.W. (2015). High efficiency, narrow particle size distribution, sub-2 μm based macrocyclic glycopeptide chiral stationary phases in HPLC and SFC. Anal. Chim. Acta.

[23] Chankvetadze B., Kartozia I., Yamamoto C., Okamoto Y. (2002). Comparative enantioseparation of selected chiral drugs on four different polysaccharide-type chiral stationary phases using polar organic mobile phases. J. Pharm. Biomed. Anal..

[24] Singh R.P., Fulton B.B., Phan H., Gout D., Lovely C.J. (2020). Ene-reaction of pre-aromatic heterocycles–thiazoles and oxazoles. Tet. Lett..

[25] Berthod A. (2006). Chiral Recognition Mechanisms. Anal. Chem..

